# Fill in the blank for fashion complementary outfit product Retrieval: VISUM summer school competition

**DOI:** 10.1007/s00138-022-01359-x

**Published:** 2022-12-30

**Authors:** Eduardo Castro, Pedro M. Ferreira, Ana Rebelo, Isabel Rio-Torto, Leonardo Capozzi, Mafalda Falcão Ferreira, Tiago Gonçalves, Tomé Albuquerque, Wilson Silva, Carolina Afonso, Ricardo Gamelas Sousa, Claudio Cimarelli, Nadia Daoudi, Gabriel Moreira, Hsiu-yu Yang, Ingrid Hrga, Javed Ahmad, Monish Keswani, Sofia Beco

**Affiliations:** 1 INESC TEC, Porto, Portugal; 2 FARFETCH, Porto, Portugal; 3 Interdisciplinary Centre for Security, Reliability and Trust, Luxembourg University, Kirchberg, Luxembourg; 4 Language Technologies Institute, Carnegie Mellon University, Pittsburgh, Pennsylvania, USA; 5 University of Stuttgart, Stuttgart, Germany; 6 Juraj Dobrila University of Pula, Pula, Croatia; 7 Visual Geometry and Modelling (VGM) Lab Italian Institute of Technology (IIT), Genova, Italy; 8 Universita degli studi di Genova, Genova, Italy; 9 Indian Institute of Technology, Hyderabad, India; 10 University of Porto, Porto, Portugal

**Keywords:** Image retrieval, Summer school competition, Computer vision, Deep learning, Fashion intelligence

## Abstract

Every year, the VISion Understanding and Machine intelligence (VISUM) summer school runs a competition where participants can learn and share knowledge about Computer Vision and Machine Learning in a vibrant environment. 2021 VISUM’s focused on applying those methodologies in fashion. Recently, there has been an increase of interest within the scientific community in applying computer vision methodologies to the fashion domain. That is highly motivated by fashion being one of the world’s largest industries presenting a rapid development in e-commerce mainly since the COVID-19 pandemic. Computer Vision for Fashion enables a wide range of innovations, from personalized recommendations to outfit matching. The competition enabled students to apply the knowledge acquired in the summer school to a real-world problem. The ambition was to foster research and development in fashion outfit complementary product retrieval by leveraging vast visual and textual data with domain knowledge. For this, a new fashion outfit dataset (acquired and curated by FARFETCH) for research and benchmark purposes is introduced. Additionally, a competitive baseline with an original negative sampling process for triplet mining was implemented and served as a starting point for participants. The top 3 performing methods are described in this paper since they constitute the reference state-of-the-art for this particular problem. To our knowledge, this is the first challenge in fashion outfit complementary product retrieval. Moreover, this joint project between academia and industry brings several relevant contributions to disseminating science and technology, promoting economic and social development, and helping to connect early-career researchers to real-world industry challenges.

## Introduction

The project competition of the 2021 VISUM^1^ summer school by INESC TEC^2^, a private non-profit research association, was co-organized with **FARFETCH**^3^, a leading company in high-fashion online marketplaces. High-fashion marketplaces require top-class customer interaction. Within this scenario, retrieving and recommending complementary product items at the right moment is a major factor in the customers’ purchasing decisions. This is particularly important in the fashion luxury domain, where customers are frequently looking for fashion items that can go well with already selected/purchased items.

The increasing demand for fashion complementary product recommendation has motivated the development of several techniques that can determine the compatibility between fashion products, through pairwise compatibility [1, 2], or outfit compatibility [3–7]. The former considers a fashion product as a query and then tries to retrieve compatible items, typically from different categories (*e.g., find a t-shirt that fits well a given pair of shoes*). The latter aims to find compatible fashion product(s) to form or complete an entire outfit.

Even though significant efforts have been made toward developing robust complementary product retrieval and recommendation techniques over the past years, fashion complementarity modeling is still a difficult task. A major challenge is related to the concept of “complementary,” which extends across different product categories. This means that compatible products, which have to be consistent in style, might be visually dissimilar. Multiple factors can define fashion compatibility, such as category, brand, color, visual appearance, material, length, among others. Additionally, although previous works report results based on open-source datasets [3, 4], to the best of our knowledge, there are no competitions in the field through which researchers can develop and compare the performance of their models using the same resources and evaluation conditions.

In this regard, the ambition of the 2021 VISUM competition is to foster research and development in the complementary product retrieval field by leveraging the vast visual and textual data together with fashion domain knowledge. Specifically, the underlying idea of the challenge is to solve the fill-in-the-blank (FITB) problem [3]. That is, given a subset of product items in an outfit and a set of candidate products from the missing category (i.e., one positive and three negatives), the task is to retrieve the most compatible candidate.

In order to support the development of the participants, we implemented and made available a baseline model. This approach follows the paradigm of distance metric learning to learn an embedding space where complementary products are closer, and non-complementary products are distant. In practice, learning distance or similarity metrics between complementary products usually resort to Siamese neural networks or triplet strategies [4, 7]. A major issue across existing approaches, which use a triplet loss to learn feature embeddings for complementary product retrieval, is the triplet generation process, which often results in the sampling of many false negative examples. A new sampling strategy was adopted based on the Louvain [8] communities of the product graph, leading to higher accuracies in the FITB problem.

This joint effort between academia and industry enabled the rapid growth and development of the participating teams when faced with a complex real-world problem. Many teams improved on the baseline solution by focusing on different aspects of the algorithm. The organization’s inference system enabled a fair evaluation of different methods, therefore validating different approaches in the area. This work focuses on the format, the main results, and the benefits to the community. The relevance of the project that originated this manuscript is thus twofold. On the one hand, it has the following main scientific contributions:

- To the best of our knowledge, this is the first fashion complementary outfit retrieval challenge, enabling an independent evaluation of different strategies for the FITB problem;
- We propose a novel negative sampling process that partially mitigates the problem of sampling false negative products - a common issue in complementary product retrieval models based on triplet strategies. This ensures a more competitive baseline model and, hence, a more challenging and appealing competition for the participants;
- A new fashion outfit dataset is introduced, which almost doubles the size of the publicly available Polyvore datasets [3, 4]. It is composed by the **FARFETCH** knowledge base and made available upon request for research and benchmark purposes. The necessity for larger-scale datasets in the fashion domain has been previously identified by Cheng et al. [9].

On the other hand, the competition impacts the community in the following ways:

- It provides an opportunity for researchers with diverse but interconnected interests to work toward a common goal, and for students to be involved in Research and Development (R &D) throughout their academic careers.
- It fosters the interest in the problem of complementary outfit retrieval and attracts more talent to work on this problem.
- It enables competence accumulation that can be transformed into value and opportunities for organizations outside the scope of the 2021 VISUM challenge.
- It establishes a partnership between a research institute and a company.

The baseline code, along with other relevant files for the competition, is publicly available^4^.

### Related work

In the following subsections, the existing datasets and competitions in intelligent fashion research are presented. In addition, a literature review of the most relevant complementary product retrieval works is also provided.

#### Datasets and competitions

According to the most recent work proposed in [9], the score of the intelligent fashion research can be categorized into four main scientific topics: 1) Fashion Synthesis, 2) Fashion Detection, 3) Fashion Recommendation, and 4) Fashion Analysis. For each, different problems are tackled, and different benchmark datasets are proposed. Fashion synthesis encompasses style transfer, pose transformation, and physical simulation. The most recent benchmark datasets that can be used in this topic are the Makeup-Wild [10], the Video Virtual Try-On [11], the DeepFashion3D [12], and the Sizer [13]. In fashion detection, problems such as landmark detection, fashion parsing, or item retrieval are included. The main benchmark datasets are the DeepFashion [14], the Fashion Landmark Dataset [15], the LIP Dataset [16], and the Amazon Dataset [17]. The line of research in fashion recommendation relies on works based on fashion compatibility, outfit matching, or hairstyle suggestion. In this manner, the PolyVore-T [18], the POG [19], and the Hairstyle30k [20] are the most common datasets. Last but not least, the three fields of focus of the fashion analysis topic are attribute recognition, style learning, and popularity prediction with the following examples of benchmark datasets: the CatalogFashion-10x [21], the FashionKE [22], and the SMPD2019 [23]. The scope of the project discussed in this paper is on the fashion recommendation, precisely on the FITB problem. Thus, a new database was created by **FARFETCH** and described in Subsect. 2.1 since the publicly available datasets [3, 4] comprise a relatively small number of annotated outfits. Additionally, the **FARFETCH** dataset contains more detailed and more expressive fashion product descriptions so as to present and cover the fine-grained attributes of the fashion items (see Table 1).

One of the existing challenges in computer vision for fashion was for text-to-image generation presented in the Workshop on Computer Vision for Fashion, Art, and Design^5^ at the European Conference on Computer Vision (ECCV) 2018. The first edition of “AI Meets Beauty” Challenge^6^, which explores fashion item recognition methods, was held in ACM Multimedia 2018^7^. To the best of our knowledge, the 2021 VISUM challenge was the first competition that introduced a fashion complementary outfit retrieval challenge to the scientific community.

#### Complementary product retrieval methodologies

In recent years, a wide range of research work has targeted the fashion complementary product retrieval problem. Existing approaches can be broadly divided into two main groups, namely (i) *pairwise compatibility* [1, 2], where complementary products are retrieved or recommended based on item-to-item compatibility, and (ii) *outfit compatibility* [3–7], where fashion complementary is addressed at the entire outfit level.

Regarding pairwise product compatibility, the authors of [1, 2] resorted to Siamese Networks to embed product images of different categories into a common feature space, referred to as the style space, in which compatible products are close to each other and products that do not fit are far apart. The embedding space was learned using a large database of product co-purchase information, which comprises product images, category labels, and their co-occurrences. From these data, *positive* (similar style) and *negative* (dissimilar style) pairs of products are strategically sampled for training. However, these methods cannot explicitly deal with entire outfit compatibility. To address this issue, many recent works [3–5, 7] have resorted to the Polyvore Outfit dataset [4] to develop truly outfit-level complementary product retrieval approaches. As an example, Han et al. [3] employed a Bi-LSTM model to learn relationships among fashion items in a given outfit. By considering an outfit as a sequence of fashion items (*e.g., * jumper, coat, skirt, shoes, sunglasses), a Bi-LSTM model is then trained to sequentially predict the next item conditioned on previously seen items and vice versa. However, considering an outfit as an ordered sequence of products poses unrealistic restrictions since product permutations in an outfit should not affect their compatibility. More recently, transformer-based architectures have emerged as alternative sequence-to-sequence approaches to LSTMs [6, 24]. Chen et al. [6] trained a BERT model to predict the masked product item in the outfit. Product compatibility is then modeled at the entire outfit level by the self-attention mechanism of the transformer. Additionally, by removing the position embedding of the transformer architecture, they are able to treat a given outfit as a set instead of a sequence of products with position information.

A major drawback across the aforementioned works is that product compatibility matching is typically performed in a single embedding space. Fashion products are comparable along multiple attribute dimensions, such as brand, category, material, color, or pattern. Dealing with this variety is also essential for robust complementary product retrieval. Bearing this in mind, several recent approaches [4, 5, 7] tried to learn multiple style subspaces to capture different notions of complementary. Vasileva et al. [4] learned a total of 66 pairwise category-specific subspaces, each one for a possible pair of product categories (*e.g., * tops-to-bottoms, tops-to-shoes, bottoms-to-shoes, etc.). These category-aware embeddings were trained with the triplet loss, where the *anchor* and *positive* samples are from different categories and appear together in an outfit, whereas a *negative* image is randomly sampled from the same semantic category as the *positive* image. In this setting, the triplet loss just operates on a single item for outfit compatibility prediction. In contrast, Lin et al. [7] proposed a novel outfit ranking loss that leverages the item relationships in an entire outfit by considering the similarities among all existing items. However, a major issue across these triplet strategies is related to the *negative* mining process since there are no manually annotated *negatives* for each outfit in the training set. By just constraining a *negative* product to be from a different outfit than the *anchor*-*positive* pair, there is a chance of sampling a large number of *false negative products* (i.e., *negatives* that can go well with the *anchor*), especially when the outfits in the training dataset share a large number of products. The baseline model of the 2021 VISUM challenge mitigates this problem by additionally constraining the *negative* sampling process with the product communities extracted from the products’ graph.

## The 2021 VISUM challenge

The 2021 VISUM challenge consisted of a five-day competition where participants designed algorithms for complementary outfit retrieval. The organization provided each team with technical support, guidance, and resources in data, code, and computational power to encourage rapid progress. Participation happened in parallel with other VISUM activities, promoting a rich environment for development. All participants had to sign a non-disclosure agreement before accessing the provided resources. We now describe the competition format and resources.

### Data

**Table 1:** Example of an outfit and the information related to it

Outfit ID	Product ID	Product Image	Product Name	Product Category	Product Description
1	000,001		Cashmere knit trackpants	Trousers	Black cashmere knit trackpants from BARRIE featuring drawstring fastening waist, slip pockets to the sides, tonal stitching, ribbed detailing and elasticated cuffs.
	000,002		Floral embroidered V-neck jumper	Knitwear	Grey cashmere floral embroidered V-neck jumper from BARRIE featuring V-neck, floral embroidery, long sleeves and ribbed detailing.
	000,003		Silk shirt	Tops	White silk shirt from SAINT LAURENT.

The *initial* dataset^8^ put together at **FARFETCH** comprises 128 398 outfits, which almost doubles the total number of outfits of the publicly available Polyvore datasets [3, 4]. Each outfit comprises an arbitrary number of products, ranging from 2 to 14 products per outfit, each containing rich multimodal information such as product image, name, category, and description. Table 1 depicts an example of an outfit along with the product information that accompanies it.

The dataset is arranged in two tabular files as follows:

- **outfits**—relates every outfit along with the corresponding set of products that belong to it, and it contains the following fields:
  - “outfit_id”: the outfit ID;
  - “main_product_id”: the main product ID, representing the anchor product in the outfit;
  - “outfit_products”: the set of product IDs that belong to the outfit.
- **products**—relates every product metadata available in the outfits table along with the required product information and is organized as follows:
  - “productid”: the product ID;
  - “productname”: the product name;
  - “category”: the product category;
  - “description”: the product description.
  Note that product images are available in a dedicated folder and named according to the corresponding product ID.

Given the competition’s short time, we selected a subset of this data, avoiding long training and inference times for the participants. We only included products that appeared at least in three outfits and belonged to the 50 larger categories. This yielded 8563 outfits, divided into two sets, *development* (5996), available to participants, and *evaluation* (2567), which were kept private at all times. This simplification ensures that rarer products and categories are excluded, but less data is available for model optimization. Table 2 provides a summary of the *initial* and *VISUM* datasets.

**Table 2:** Summary of *initial* and *VISUM* datasets

product’s representation	- image; - name; - category; - description.	
Sets	Initial	VISUM
# outfits	128,398	8563
# unique products	149,944	8129
# prod./outfit (min/max/avg)	2/14/4.5	2/10/3.9
# categories	133	50

Throughout the rest of the paper, we will use the following notation: let ${\varvec{X}}^{(l_i)}_{o_i,c_i}$ and ${\varvec{T}}^{(l_i)}_{o_i,c_i}$ denote the image and the textual description of a given product item *i*, where $o_i$, $c_i$, and $l_i$ represent the corresponding outfit ID, product category, and product community, respectively. As detailed in Sect. 2.3.2, product communities are obtained through the Louvain method on the products’ graph, which will be used to constrain the sampling process.

### Competition format

The teams were asked to solve the FITB problem; given an incomplete outfit (query) and a set of candidate products, the algorithm should return the candidate that completes the outfit. For each query, there are four candidates of the same product category, of which only one is considered complementary (i.e., positive). As an additional restriction, the algorithm should run in an acceptable time (less than one hour for inference in the evaluation set).

To generate the test queries, for each outfit: i) one random product was removed and added to the candidate set as positive; and ii) three products were sampled from the whole set, belonging to the same category as the positive, and added to the candidates as negatives. Accuracy was selected as the evaluation metric to compare solutions and defined as:

1 $$\begin{aligned} \text {Acc} = \frac{1}{N}\sum _{i=1}^{N} {\mathbb {I}}_{[{\hat{y}}_i = y_i]}, \end{aligned}$$

where ${\hat{y}}_i$ and $y_i$ denote the predicted and ground truth candidate products, respectively. *N* is the number of queries, and ${\mathbb {I}}_{[{\hat{y}}_i = y_i]}$ is an indicator function, which equals to 1 whenever ${\hat{y}}_i=y_i$, and 0 otherwise.

Each participating team was provided with a virtual machine for the period of the competition, guaranteeing a standard level of processing power for all. Participants could access the challenge data and baseline code within this machine. The baseline code already followed the defined evaluation protocol for the challenge. This greatly reduced the setup time necessary to start working on a solution. Table 3 shows the specifications of these virtual machines.

**Table 3:** Description of the virtual machine provided to the participants

Component	Description & Properties
CPU	$4 \times$ vCPUs (Intel Broadwell)
GPU	$1 \times$ NVIDIA Tesla T4
Storage	60GB
RAM	15GB

For evaluation, we followed a model-to-data approach, ensuring that participating teams did not, at any point, have access to the evaluation set. For this, a web application was set up, where participants could request a *submission*. Upon this request, the organization’s server would access the participants’ virtual machine, copy its contents and run their algorithm locally against the private data. The resulting accuracy would then be displayed on a public leaderboard accessible to all teams.

**Fig. 1:**
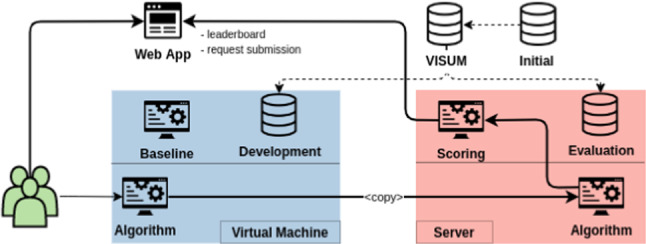
**Competition Diagram**: The blue area corresponds to the virtual machine to which participants had access. The red area was kept private. The highlighted path indicates the flow of information after a participant requests a submission

During the competition, teams were allowed to request submissions up to three times per day. These results were merely indicative, and the accuracy was estimated using a fixed subset (referred to as the *daily test*) of the test queries corresponding to around 30% of the whole test set. The final leaderboard was computed using all test queries after the competition deadline. This final leaderboard was used to decide the winner. Figure 1 summarizes the framework used to organize the competition.

### Baseline

The baseline model is trained to map the product image and description into a common multimodal “complementary” embedding space in which compatible products are close to each other, and non-complementary products are far apart. For inference, the distances between the outfit products and the candidates are used to select the most compatible candidate.

The work of Lin et al. [7] and Vasileva et al. [4] are the most related to our proposed baseline. Nevertheless, in terms of triplet strategies, our proposed negative sampling process mitigates the problem of sampling false negative products, which is a common issue in these state-of-the-art approaches.

#### Architecture

**Fig. 2:**
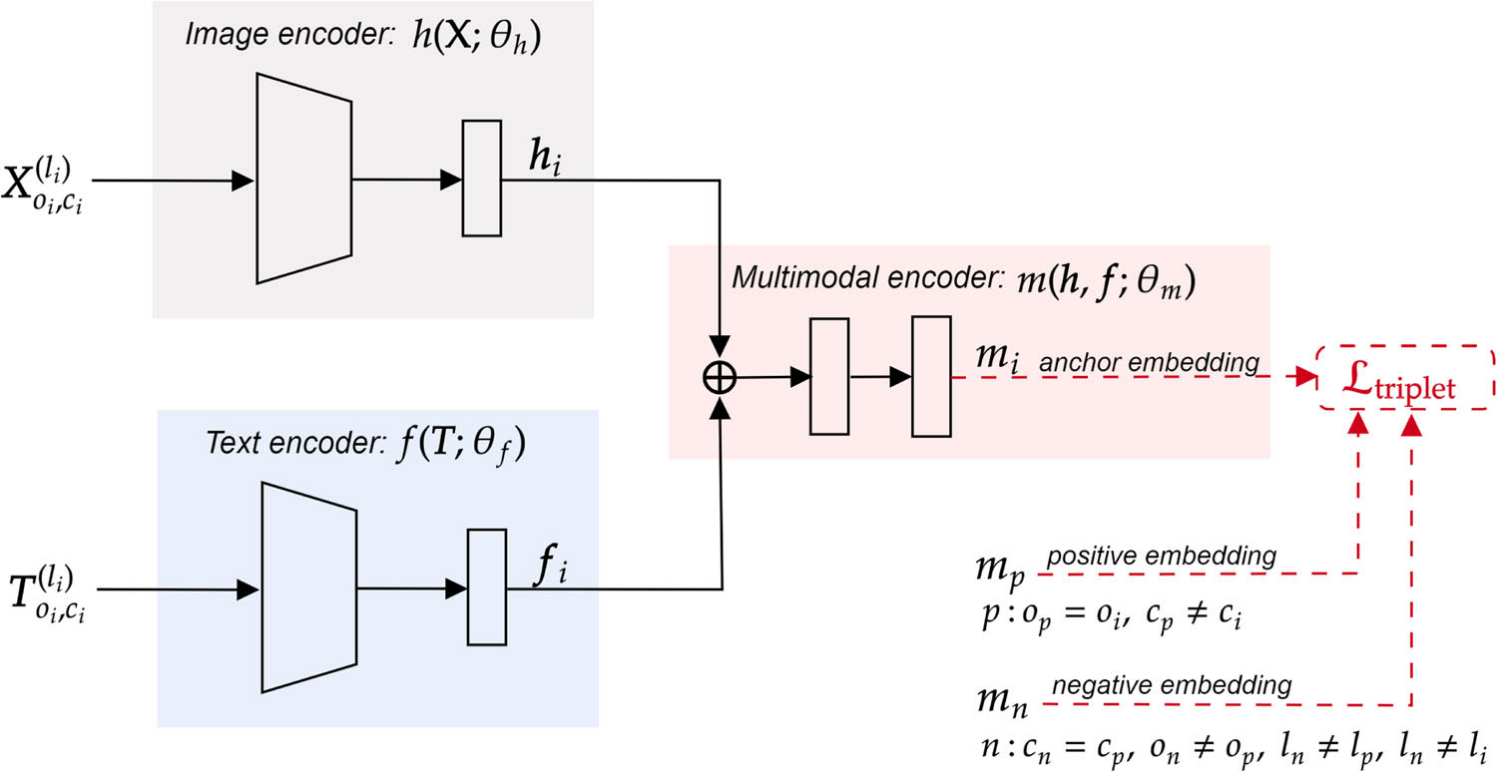
Architecture of the baseline model. It comprises three main sub-networks or blocks, i.e., an *image encoder*, a *text encoder*, and a *multimodal encoder*, whose parameters are jointly optimized via a triplet loss

To induce the model to learn a complementary embedding space, the implemented baseline model comprises three main modules or sub-networks (see Figure 2): an *image encoder*, a *text encoder*, and a *multimodal encoder*.

***Image encoder*** The *image encoder* aims at learning an encoding function $h({\textbf{X}};\theta _h)$, parameterized by $\theta _h$, that maps from an input product image ${\textbf{X}}$ to a latent feature representation ${\varvec{h}}$.

The architecture of the image encoder comprises a pre-trained ResNet-50 [25] as its base block, due to its widespread adoption and proven performance, followed by a projection block with additional trainable layers to increase the overall representational capability of the encoder for our task. The projection block consists of two fully connected layers, with the first one having a Gaussian error linear unit (GELU) nonlinearity, a dropout layer, and a residual connection between the first and last layers. In order to maintain feature comparability, the output image representation ${\varvec{h}}$ is normalized onto the unit hypersphere, i.e., $\Vert {\varvec{h}}\Vert =1$.

***Text encoder*** The purpose of the *text encoder* is to learn an encoding function $f({\varvec{T}};\theta _f)$, parameterized by $\theta _f$ that maps from a given product description ${\varvec{T}}$ to a latent text representation ${\varvec{f}}$. It consists of a pre-trained DistilBERT model [26] followed by a projection block with the same topology as the image encoder. Following the original BERT and DistilBERT papers [26, 27], the hidden representation of the classification (CLS) token is used to summarize the whole product description. This works under the assumption that this representation is able to capture the overall meaning of the product description. For feature comparability, $l^2$ normalization is also applied to the output text embedding ${\varvec{f}}$.

***Multimodal encoder*** Lastly, the *multimodal encoder* $m({\varvec{h}},{\varvec{f}};\theta _m)$ attempts to learn a mapping from both visual and text representations, ${\varvec{h}}$ and ${\varvec{f}}$, to a multimodal “complementary” feature space. The multimodal encoder comprises a merge layer that first concatenates both text and image representations, followed by a projection block (with the same topology as the other two encoders) to properly fuse both modalities into a shared embedding space. The final multimodal latent representation ${\varvec{m}}$ is also normalized onto the unit hypersphere.

#### Training

The network parameters are optimized via a ranking loss, i.e., triplet loss that forces the distance between non-complementary product embeddings the (*anchor* and *negative* samples) to be larger than the distance of complementary product embeddings (*anchor* and *positive* samples) by a margin $\alpha$.

Formally, let $m^{(l_i)}_{o_i,c_i}$ be an anchor’s multimodal latent representation, and $m^{(l_p)}_{o_p,c_p}$ and $m^{(l_n)}_{o_n,c_n}$ represent positive and negative multimodal representations, respectively. The triplet loss ${\mathcal {L}}_{\text {triplet}}$ used to train the implemented baseline is defined as follows:

2 $$\begin{aligned}&{\mathcal {L}}_{\text {triplet}} =\frac{1}{N}\sum _{i=1}^{N} \max \nonumber \\&\left( \left[ \Vert m^{(l_i)}_{o_i,c_i}-m^{(l_p)}_{o_p,c_p}\Vert ^2 - \Vert m^{(l_i)}_{o_i,c_i}-m^{(l_n)}_{o_n,c_n}\Vert ^2 + \alpha \right] , 0\right) , \end{aligned}$$

where $o_p=o_i$ and $c_p\ne c_i$, which means that the *anchor* and *positive* samples are from different categories and appear together within the same outfit. Additionally, $c_n=c_p$, $o_n\ne o_p$, $l_n\ne l_p$, $l_n\ne l_i$, which means that the mining process of negative samples is being constrained not only by the product category and outfit ID (as in previous works [4, 5, 7]), but also on the product communities. That is, a *negative* is randomly sampled from a different outfit but with the same semantic category as the *positive*, and further constrained to belong to a different product community. The underlying idea of applying the community constraint is to further reduce the likelihood of selecting *negatives* that can go well with the *anchor* (i.e., *false negatives*). This is increasingly relevant when the outfits in the training dataset share a large number of products.

Summing up, at each training iteration, we sample a mini-batch of *N* triplets according to the following constraints:

- *positive* and *anchor* pairs have to belong to the same outfit but from a different category $(o_p=o_i, c_p\ne c_i)$;
- *positive* and *negative* pairs have to belong to the same semantic category $(c_p=c_n)$;
- *positive* and *negative* pairs have to belong to different outfits $(o_p\ne o_n)$;
- *positive* and *negative* pairs have to belong to different communities $(l_p\ne l_n)$;
- *anchor* and *negative* pairs have to belong to different communities $(l_i\ne l_n)$.

**Fig. 3:**
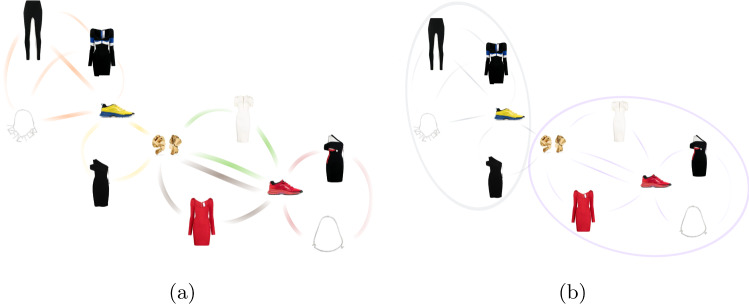
Product communities detection: (a) products’ graph built from 5 outfits, where each color represents an outfit; and (b) the two detected Louvain communities, each one delineated by an ellipse marked at a different color

To apply the above-mentioned community constraints, we resort to the Louvain method, which connects communities by optimizing the modularity of the products’ graph, which measures the relative density of edges inside the communities concerning the outside edge. The nodes in the products’ graph denote the products, while the edges represent the number of occurrences between two products in outfits. Figure 3 depicts the product communities detection process. In this particular example, two communities were found from a total of five outfits, which reduces the likelihood of sampling *false negatives* during training.

#### Inference

After training, model inference can be simply performed by querying the learned “complementary” embedding space to return the most compatible product. The model receives a query representing the outfit and a set of candidates composed of four products (one positive and three negatives). Then, the predicted candidate corresponds to the candidate with the lowest sum of the distances to all the query products. Formally, let *Q* be the query outfit and *C* the set of four candidates. Then, prediction ${\hat{y}}$ is defined as:

3 $$\begin{aligned} {\hat{y}} = {{\,\mathrm{arg\,min}\,}}_{\text {cand} \in C} \sum _{\text {query} \in Q} \Vert m_\text {query} - m_\text {cand} \Vert ^2 \end{aligned}$$

## Results and discussion

### Baseline analysis

This section presents an analysis of the implemented baseline on the *initial* dataset. In this regard, Table 4 depicts the impact of each data modality and the additional Louvain constraint in the overall FITB test accuracy.

**Table 4:** Experimental results of different versions of the implemented baseline model on the test of the *initial* dataset

Modality	FITB accuracy	
	w/ Louvain	wo Louvain
Image	0.454	0.442
Text	0.471	0.433
Multimodal (Image + Text)	**0.483**	0.472

All versions of the baseline were trained for 100 epochs using the Adam optimization algorithm with a learning rate of $1e^{-3}$, a batch size of 128 triplets, and a margin $\alpha$ of 1.0. Regarding regularization techniques, the $l^{2}$ coefficient was set to $1e^{-4}$, and the dropout rate was empirically set as 0.1. In terms of model architecture, we used an embedding size of 2048 for the image encoder, 768 for the text encoder, and 1024 for the multimodal encoder. The text descriptions were tokenized with the DistilBERT tokenizer from the HuggingFace^9^ library to properly feed the token IDs and the attention masks to the DistilBERT model.

Regarding the obtained results, the most interesting observation is that the multimodal version of the implemented baseline, along with the proposed Louvain constraint for negative sampling, leads to the overall best results (i.e., 0.483). These results attest to both: (i) the importance of both textual and visual cues for retrieving complementary products and (ii) the benefits of the Louvain constraint for reducing the likelihood of sampling false negative products during training.

Based on these results, the top performing version (Multimodal w/ Louvain) was adopted as a baseline for the challenge. A new model was optimized using the *development* set for 30 epochs after verifying that training further resulted in no additional gains in terms of validation accuracy. The accuracy of the generated test queries for this model was 0.426. The drop in accuracy can be explained by the much smaller size of this subset of data when compared to the *initial* dataset.

Finally, as a post-event evaluation, we compared the baseline model with the state-of-the-art works in the field on the well-known Polyvore Outfits-D (disjoint) dataset ( [4]). A total of around 32k outfits are available. The standard train/validation/test split was followed, as in the original article. Optimization was done for 30 epochs after verification that this was enough for model convergence. Results are depicted in Table 5.

**Table 5:** Comparisson of the baseline model with state-of-the-art methods on the Polyvore Ourfits-D dataset

Method	Image	Text	FITB accuracy (%)
Siamese-Net [4]	$\checkmark$		51.80
Type-aware [4]	$\checkmark$	$\checkmark$	55.65
SCE-Net average [5]	$\checkmark$	$\checkmark$	53.67
CSA-Net (triplet loss) [7]	$\checkmark$		56.17
CSA-Net (outfit ranking loss) [7]	$\checkmark$		59.26
OutfitTransformer [28]	$\checkmark$	$\checkmark$	59.48
Baseline (ours)	$\checkmark$	$\checkmark$	54.20

Conceptually, our approach is similar to the Siamese Network developed in [4], with the added benefit of being multimodal and using an enhanced sampling method based on the Louvain communities. These differences justify the increased accuracy when compared to this simple solution. Our baseline is outperformed by more competitive approaches, such as the OutfitTransformer [28] and the CSA-Net (using outfit ranking loss) [7]. These recent methods introduce changes to the model architecture and loss function, which address challenges in the problem of complementary item retrieval and are not present in the implemented solution. Interestingly, as shown in sect. 3.3, some participating teams introduced ideas from [7] into their approach, which led them to higher FITB accuracies. This constitutes an external validation of the state-of-the-art in a new dataset.

### Challenge results

A total of 52 people participated in the competition, corresponding to 60% of the total number of students in the summer school. Most participants were either PhD students or recently graduated (less than five years) professionals with previous experience in machine learning or computer vision. In total, 20 different nationalities of affiliation were represented. Most of these were from Europe with the following exceptions: Brazil, the USA, India, South Africa, and Pakistan. Women constituted 35% of the participants.

**Fig. 4:**
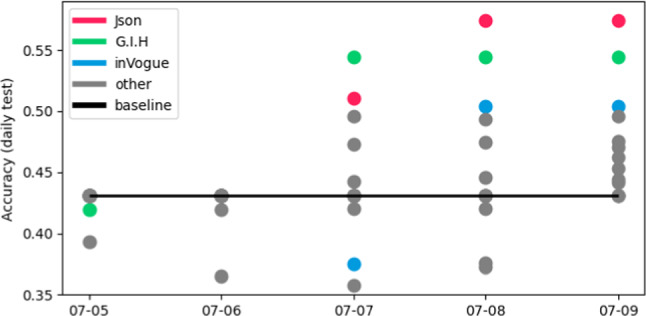
Accuracy on the *daily test* of the best submission for different teams throughout the competition duration. The top-3 performing teams are highlighted. An absent marker means the team did not submit any algorithm that day. Notice that improvements started appearing later in the competition

Participants formed into 18 teams, out of which 16 made at least one valid submission throughout the five-day competition and participated in two brainstorming sessions with computer vision experts, including professionals working in automatic complementary product retrieval 10. Teams collectively submitted 120 algorithms for evaluation, of which 32 (27%) were better than the baseline on the *daily test* set. This low percentage is explained by an initial learning phase, where participants focused on problem understanding and getting used to the baseline code and submission process. Generally, in the first days, teams performed worse or equal to the baseline, while later, a majority of teams were able to surpass it, as shown in Figure 4. The first solution to surpass this threshold happened on the third day of the competition. In the final submission, 10 teams (56%) outperformed the baseline. The final leaderboard is presented in Table 6.

Regarding methodology, all teams that were able to surpass the baseline accuracy based their solution, at least partially, on the baseline provided. Generally, teams resorted to learning deep multimodal product encoders, which returned embeddings in which complementary items are close, and non-complementary are far apart. Teams improved on the baseline by innovating on aspects such as encoder architecture, modality fusion, ensembling, use of different loss functions, and training settings. The three top-performing solutions are described later in sect. 3.3. Notably, among the literature, the works of [7] and [4] played a bigger role since they were discussed during the brainstorming sessions.

**Table 6:** Evaluation accuracy achieved by the participants in the challenge (calculated on the *complete test* set). The results for the 8 teams that scored lower or equal to the baseline are omitted

Team	Accuracy
Json	0.54538
G.I.H	0.52279
inVogue	0.51110
Gucci-vision	0.50292
DeepVis	0.48929
Clothing objects REcommender System (CORES)	0.47565
DataSense	0.46474
Next top model	0.46358
The fledglings	0.44137
Um due tres	0.44098
(Baseline accuracy)	0.42579

The aggregate results illustrate the difficulty of the competition. This can be attributed to different factors: i) the existence of a competitive baseline; ii) the short time span of the challenge; and iii) the relatively steep learning curve of the problem. Participant feedback emphasized the two latter factors described. Despite this, most teams improved on the baseline, with the top teams achieving a considerable margin.

### Top-3 teams

**Fig. 5:**
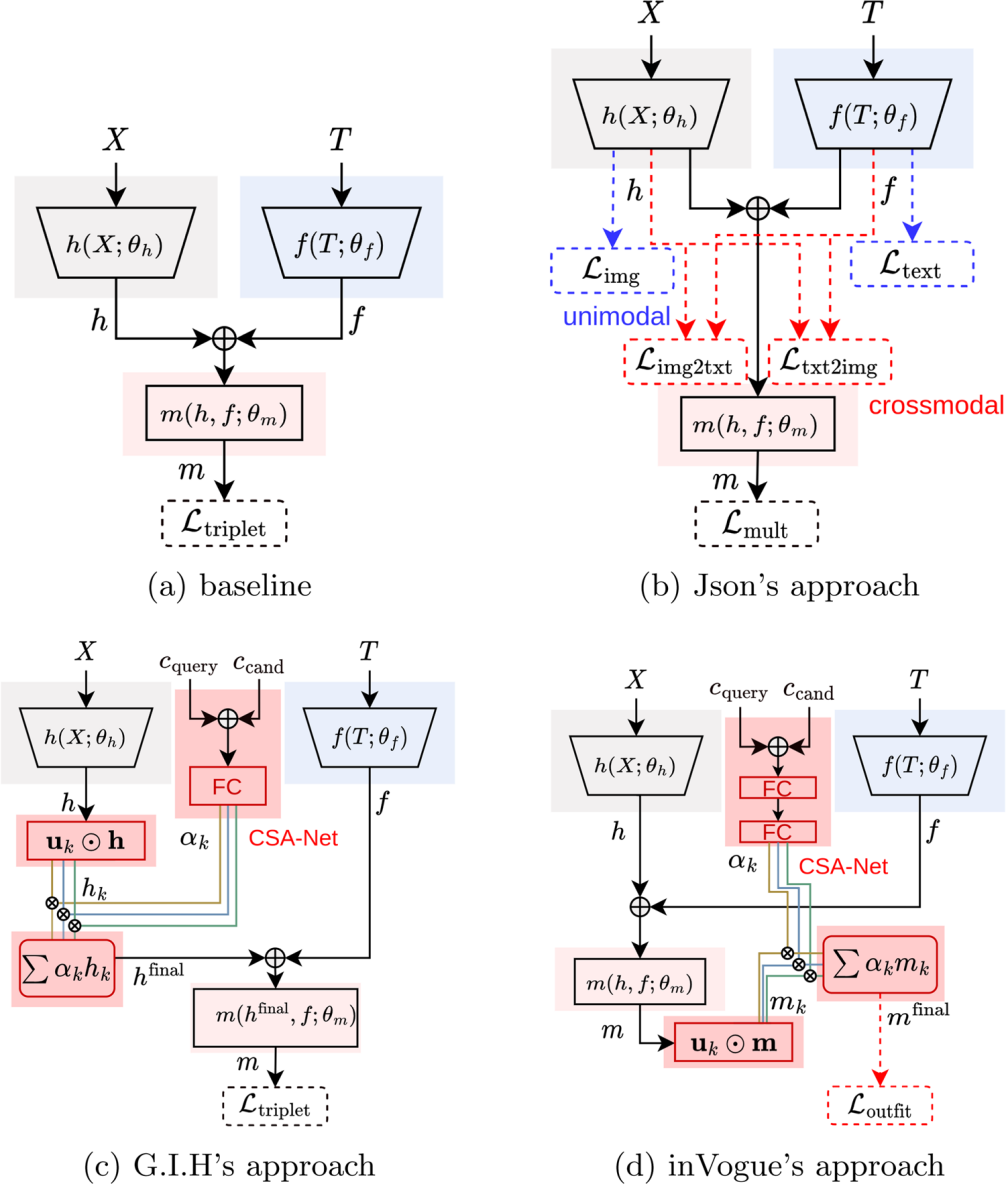
Diagrams of all the algorithms used in this work. Stronger colors are used to highlight the changes proposed by the participants in their approach

This subsection presents the approaches of the three top-performing teams: “Json”, “G.I.H” and “inVogue.” Similar to the baseline solution, all three use encoders to learn the complementary relations. These encoders map products to an embedding space where complementary items are close and non-complementary are far apart. For inference, the average distance to the items in the query outfit is used to select the most complementary candidate.

The three teams introduced variations into the baseline solution, improving accuracy on the private test set. Json’s approach (first place) followed an ensemble strategy based on the multiple embedding spaces returned by the three encoders, ${\varvec{h}}$, ${\varvec{f}}$, and ${\varvec{m}}$, and is described in sect. 3.3.1. The approaches proposed by “G.I.H” and “inVogue” partially overlap in the use of category-based subspace attention networks (CSA-Nets), initially proposed by [7] and are described in 3.3.2 and 3.3.3, respectively. To highlight the different approaches, Figure 5 compares all the algorithms used in this work (including the baseline).

#### “Json”

The following method was submitted by the “Json” team composed of Javed Ahmad, Sofia Beco, and Nadia Daoudi. The proposed methodology follows the diagram in Figure 5b.

In the baseline solution, the triplet loss is used to learn a multimodal complementary embedding space, which is useful for retrieval. The image and text encoders, ${\textbf{h}}$ and ${\textbf{f}}$, are only used as intermediate representations to obtain the multimodal representation ${\textbf{m}}$. In Json’s approach, these embeddings are also used for learning and inferring complementary relations. To the best of our knowledge, this is an innovation over the state-of-the-art.

Instead of using only the multimodal triplet loss (here denoted as ${\mathcal {L}}_\text {mult}$) defined in Equation 2, we introduce additional unimodal and crossmodal loss functions. For this, the image and text embeddings are fed into the triplet loss as anchors, positives and negatives. A summary of the five losses considered is shown in Table 7.

**Table 7:** Losses used during optimization by the Json team. The best weights column regards the weighting of the different losses in the second training phase

Loss	Type	Anchor modality	Pos / Neg modality	Best weights
${\mathcal {L}}_{\text {img}}$	Unimodal	Image	Image	1
${\mathcal {L}}_{\text {txt}}$		Text	Text	0
${\mathcal {L}}_{\text {img2txt}}$	Crossmodal	Image	Text	0
${\mathcal {L}}_{\text {txt2img}}$		Text	Image	0
${\mathcal {L}}_{\text {mult}}$	Multimodal	Multimodal	Multimodal	1

The final loss function is a weighted sum of the five triplet losses defined:

4 $$\begin{aligned} {\mathcal {L}} =&\, w_1.{\mathcal {L}}_{\text {img}} + w_2.{\mathcal {L}}_{\text {txt}} \nonumber \\&+ w_3.{\mathcal {L}}_{\text {img2txt}} + w_4.{\mathcal {L}}_{\text {txt2img}} \nonumber \\&+w_5.{\mathcal {L}}_{\text {mult}} \end{aligned}$$

For inference, five distances are analogously defined depending on the embedding used to encode the query and candidate products. The chosen candidate is the one that minimizes a weighted sum of these five distances:

5 $$\begin{aligned} {\hat{y}} =&{{\,\mathrm{arg\,min}\,}}_{\text {cand} \in C} \sum _{\text {query} \in Q} w_1.\Vert h_\text {cand}-h_\text {query}\Vert ^2 \nonumber \\&+w_2.\Vert f_\text {cand}-f_\text {query}\Vert ^2 \nonumber \\&+w_3.\Vert f_\text {cand}-h_\text {query}\Vert ^2 + w_4.\Vert h_\text {cand}-f_\text {query}\Vert ^2 \nonumber \\&+ w_5.\Vert m_\text {cand}-m_\text {query}\Vert ^2 , \end{aligned}$$

where *Q* denotes the set of products in the query outfit. The weights used are the same as those used during optimization to balance the different loss functions.

Two training phases were considered. Initially, all weights, $w_i$’s, were set to one and the model optimized for 63 epochs, with a batch size of 128 and a learning rate of 0.0001. After this initial step, we experimented setting the $w_i$’s to different values and performing inference on the validation data. The weight combination that yielded the best results was setting $w_1$ (${\mathcal {L}}_{img}$) and $w_5$ (${\mathcal {L}}_{mult}$) to 1 and all others to 0. A second phase of training was run, where this weight combination was also used in training. The model was fine-tuned for 82 additional epochs, using a batch size of 64.

#### “G.I.H”

The following method was submitted by the “G.I.H” team composed of Gabriel Moreira, Ingrid Hrga, and Hsiu-yu Yang. The proposed methodology follows the network architecture presented in Figure 5c.

The baseline solution was improved by considering different components of compatibility in the image representation ${\textbf{h}}$. The weight of each of these components is based on the categories of the query product and the candidate, leading to a category-aware image feature vector. This approach is based on the Category Subspace Attention Network (CSA-Net) proposed by [7].

A set of masks, ${\textbf{u}}_k$’s, are used to extract *K* components, ${\textbf{h}}_k$, from the image representation. For this the element-wise product (denoted as $\odot$) is used, such that:

6 $$\begin{aligned} {\textbf{h}}_k = {\textbf{u}}_k \odot {\textbf{h}} \end{aligned}$$

These masks are composed of learnable parameters which are optimized by backpropagation normally. They encode for different visual components which are weighted differently depending on the categories of the query and candidate products. The final image representation given by:

7 $$\begin{aligned} {\textbf{h}}^\text {final} = \sum _{k=1}^K \alpha _k.{\textbf{h}}_k \end{aligned}$$

The attention coefficients are returned by a neural network which takes as input the one-hot encoding of the query and candidate product categories and returns coefficients that sum up to one by means of a softmax activation in the output.

Optimization-wise, 5 visual components ($K=5$) were used in the CSA-Net implementation. The attention coefficients were computed by a sub-network with one fully connected layer. The whole model was trained for 40 epochs, using the Adam optimizer and with a learning rate of 0.0001. No regularization was used (dropout and $l^2$). All other hyper-parameters were the same as the baseline.

#### “inVogue”

The following method was submitted by the “inVogue” team composed of Monish Keswani and Claudio Cimarelli. The proposed methodology follows the network architecture presented in Figure 5d.

The previously described CSA-Net method was also employed by “inVogue” team but on the multimodal representation ${\textbf{m}}$, rather than the image embedding as in the original paper. That is, the final multimodal embedding ${\textbf{m}}^\text {final}$ is a weighted sum of the multimodal subspace embeddings:

8 $$\begin{aligned} {\textbf{m}}^\text {final} = \sum _{k=1}^K \alpha _k . [{\textbf{u}}_k \odot {\textbf{m}}], \qquad \end{aligned}$$

where *K* denotes the total number of multimodal subspaces, ${\textbf{u}}_k$ represents the *i*-th learnable mask, and $\alpha _k$ is the corresponding attention weight.

Additionally, the outfit ranking loss proposed in [7] was also used. Contrary to the triplet loss which uses as negative and positive one individual product, the outfit ranking loss defines a “distance to the outfit” metric (one to many) and thus the positive and negative samples are whole outfits. Specifically, during training, a positive *P* is sampled by selecting all the products in an outfit which the anchor is part of. The negative *N* is sampled as a set of products which form an outfit, but which the anchor is not present. We define the distance of a product to the entire outfit as:

9 $$\begin{aligned} d(\text {anchor}, O) = \frac{1}{\#O} \sum _{prod \in O} \Vert m_\text {anchor} - m_\text {prod}\Vert ^ 2, \end{aligned}$$

where $\text {anchor}$ is an individual product, *O* represents an entire outfit, and $\#O$ is the total number of products within outfit *O*. Notice that this distance is used to decide the most likely candidate during inference in the baseline solution (by setting *O* to the query outfit and $\text {anchor}$ to each candidate). The final loss is given by:

10 $$\begin{aligned} {\mathcal {L}}_{\text {outfit}}=&\frac{1}{N}\sum _{i=1}^{N} \max \left( 0, \left[ d(\text {anchor}, P) \right. \right. \nonumber \\&\left. \left. - d(\text {anchor}, N) + \alpha \right] \right) , \end{aligned}$$

where *P* represents a set of products from an outfit to which the $\text {anchor}$ belongs, and *N* is a set of negative products from a different outfit.

Notice that although the average is used as an aggregation function, in the original paper, this function does not necessarily take this form (e.g., min).

The final architecture included a CSA-Net with 10 visual components ($K=10$). The attention coefficients were obtained using two fully-connected layers of size 1024. The baseline weights were used for initialization of all encoders. The batch size was 128, and the learning rate was set to 0.0001. Adam was used as an optimizer. Dropout was removed and the $l^2$ coefficient was set to $1e^{-6}$. The learning rate was decreased by a factor of 10 after 10 epochs without improvement on the training loss.

### Discussion

The proposed model for the competition proved to be valuable for the participants. By providing them with resources, guidance, and a complete setup in the form of ready-to-go virtual machines, participants are at the center of the challenge, fully empowered to learn and quickly improve. This translated into high engagement levels, as shown by the number of submitted algorithms during the competition. It also enabled most teams to remain competitive, despite the limited experience of some participants, the high complexity of the problem, and the short amount of time available.

The model-to-data approach followed in the evaluation process provided a fair evaluation for all. The only information released after each submission was the accuracy obtained by the algorithm on a limited subset of the evaluation data. It is worth mentioning that, for sensible data, a similar model could be implemented, disabling participants’ access to training data as well. In this case, participants would submit training algorithms, and it would be necessary to limit the output of these to prevent data leaking.

All top-3 teams proposed creative adaptations to the baseline model, significantly improving its accuracy. The “Json” followed an ensembling strategy with models trained on different modalities. The “G.I.H” and the “inVogue” proposed learning subspace embeddings conditioned on the product category. While the “G.I.H” uses this strategy for the image representations only, the “inVogue” applies it to the multimodal representation. The “inVogue” also implemented an outfit ranking loss [7], considering all the outfit products in each training triplet, instead of randomly sampling two for positive and anchor. There were some promising but unexplored directions in this challenge. These include collaborative filtering [29], commonly used in e-commerce, which can avoid having to learn product embeddings as a whole to model the notion of product complementary. Also, all top strategies, similar to the baseline, modeled product complementarity as pairwise distances, which may be sub-optimal. In other words, two products may be complementary within the context of an outfit but non-complementary within another. Recent works in the field have addressed this differently [6].

The partnership that allowed this competition proved to be valuable at different levels. The presence of industry enables interesting, real-world problems and brings diversity and new perspectives to the set of topics typically covered in scientific challenges. The direct benefit of these goes to the participants who are enabled to try R &D in a richer environment. Also, this challenge drew the community’s attention to the problem of complementary fashion outfit retrieval, bringing new ideas and knowledge to the field, which can lead to improvements in the state-of-the-art. Finally, communication and cooperation between industry and academia help bridge the gap between research and applied research. By easing the knowledge diffusion process, organizations can better deal with current and future scientific problems and accelerate the pace at which basic research translates into technology, ultimately leading to long-term economic and social gains.

## Conclusion and future work

The 2021 VISUM project competition represented the first fashion complementary outfit retrieval challenge while simultaneously remaining a stimulating learning opportunity for its participants. The proposed competition format was effective at enabling teams to learn and improve, despite the short time span of the summer school. To this end, a competitive baseline was implemented and provided to the participants, along with the challenge’s data and computational resources. Most teams were able to surpass this baseline model. Qualitatively, the top-performing teams presented diverse approaches and were able to beat the baseline threshold by a considerable margin.

Organizing this challenge embodied a joint effort between academia and industry. Leveraging the knowledge between these two domains was vital in creating an engaging real-world problem for the community. In general, such partnerships represent the diversification of the topics covered in scientific challenges and richer environments for the interaction and learning of the participants. Finally, projects such as these contribute to filling in the gap between research and applied research by fomenting new communication and knowledge networks that ease the dissemination of results.

## A FARFETCH

**FARFETCH** is the leading global platform for the luxury fashion industry, connecting creators, curators and consumers. Founded in 2007 by José Neves for the love of fashion, and launched in 2008, **FARFETCH** began as an e-commerce marketplace for luxury boutiques around the world. Today, the **FARFETCH** Marketplace connects customers in over 190 countries and territories with items from more than 50 countries and over 1,300 of the world’s best brands, boutiques, and department stores, delivering truly unique shopping experience and access to the most extensive selection of luxury on a single platform.

## B VISUM summer school

VISion Understanding and Machine intelligence (VISUM)^11^ is a non-profit summer school that aims to gather PhD candidates, postdoctoral scholars, and researchers from academia and industry with research interests in computer vision and machine intelligence. It is organized by the Institute for Systems and Computer Engineering, Technology and Science (INESC TEC)^12^, Porto, Portugal. We, the Organizing Committee, pursue a never-ending visual information learning system, to empower the next generation of intelligent systems with the capability of reasoning from visual data. We perform research in both fundamental and applied problems in computer vision, image processing, machine learning, and decision support systems anchored in the automatic analysis of visual data. From our research interests and the passion to share knowledge, VISUM was born. In light of the Coronavirus disease (COVID-19) outbreak, the 2021 VISUM summer school was fully digital. In this manner, several platforms was provided in advance to the participants to promote the remote work. The 2021 VISUM created an unique Zoom link for all theoretical and hands-on sessions. A Discord server was used to increase the interaction between participants and guarantee more readily available support from the project staff. Furthermore, all the materials were made available in VISUM’s GITHUB page^13^.

2021 VISUM was a summer school of six days composed of both fundamental topics and more applied ones to embrace diversity in the applicants and give them different concepts in the computer vision area. Basics sessions, which occurred on the first day, comprised the following five lectures:

- Machine Learning (with Python and scikit-learn);
- Computer Vision with Deep Learning;
- PyTorch and TensorFlow;
- Introduction to Bayesian Networks.

Image Retrieval (by Prof. Henning Müller, HES-SO Valais), Image Understanding (by Prof. Hugo Proença, University of Beira Interior), Graph Neural Networks (by Prof. Michael Bronstein, Imperial College London), Causality for Machine Learning (by Prof. Jonas Peters, University of Copenhagen), and Trustworthy AI (by Prof. Peter Eisert, Humboldt University) were the topics of the remaining week. All the lectures were recorded and made available as streaming media with no download option for the entire duration of the school and the following week. In this way, we guaranteed that everyone would be able to follow all lectures, even if they were delivered at an inconvenient time for the participants.

Moreover, in order to bridge the gap between industry and academia, 2021 VISUM had a workshop on Deploying Machine Learning Models by Dattaraj Rao from Persistent^14^ and, during the coffee-breaks Kelwin Fernandes from NILG.AI^15^ talked about a) Learning spectrum, b) Embedding domain knowledge in Deep Neural Networks, and c) Self-supervised learning.

On the last day of 2021 VISUM, an international panel of experts in law and AI discussed how AI should be regulated. Some questions that were debated were a) which current problems of AI could (and should) be addressed through carefully drafted laws? b) which ethical concerns have been overlooked amidst the recent frenzy of AI and computer vision? and c) which fields of AI raise the most pressing ethical and privacy concerns?

In a nutshell, the main contributions of VISUM summer school to the computer vision community throughout these last nine years are the following:

- A summer school composed of **Theoretical and Practical lessons** on *avant-garde* **topics** in computer vision given by world-renowned experts;
- Cooperation between industry and academia with an **Industry Day** where participants have the opportunity to meet people with knowledge and experience in **Computer Vision**, expand contacts and promote discussions, networking or future collaborations;
- A **Project Competition** for the VISUM attendees to make the event even more challenging;
- **2 ECTS**^16^ to the participants of the VISUM Project Competition.

VISUM is positioning itself as a reference summer school throughout the years, with an increasing number of participants, recognition from speakers, partners and students. This edition had a total of 87 participants from 20 different countries. Definitely, VISUM has been growing not only in the number of participants, but also in terms of maturity, partnerships, and visibility inside the Computer Vision Community. VISUM is an extraordinary opportunity to be close to cutting-edge concepts and technology of machine learning and computer vision. Through the creation of a specialized multicultural environment, it is intended to promote and increase the knowledge of all participants on the state of the art of these topics, provided by internationally renowned experts in the data science field. Being an area of great potential for industrial applications with a strong increase in the number of researchers in recent years, VISUM is also an unmissable opportunity for networking and contacts exchange for everyone involved.

## Footnotes

1 https://visum.inesctec.pt.

2 https://www.inesctec.pt/.

3 https://www.farfetch.com.

4 https://github.com/visum-summerschool/visum-competition2021.

5 https://sites.google.com/view/eccvfashion/.

6 https://challenge2020.perfectcorp.com.

7 https://challenge2018.perfectcorp.com.

8 Data available upon request from the corresponding author, Pedro M. Ferreira.

9 https://huggingface.co/.

10 Regarding the two “inactive” teams, one interacted with the staff and worked on the problem but decided not to compete, while the other dropped out early on.

## References

[1] Veit, A., Kovacs, B., Bell, S., McAuley, J., Bala, K., Belongie, S.: Learning Visual Clothing Style with Heterogeneous Dyadic Co-occurrences. In: International Conference on Computer Vision (ICCV), Santiago, Chile (2015). *Equal Contribution

[2] McAuley, J., Targett, C., Shi, Q., van den Hengel, A.: Image-based recommendations on styles and substitutes. In: Proceedings of the 38th International ACM SIGIR Conference on Research and Development in Information Retrieval. SIGIR ’15, pp. 43–52. Association for Computing Machinery, New York, NY, USA (2015). 10.1145/2766462.2767755

[3] Han, X., Wu, Z., Jiang, Y.-G., Davis, L.S.: Learning fashion compatibility with bidirectional lstms. In: Proceedings of the 25th ACM International Conference on Multimedia. MM ’17, pp. 1078–1086. Association for Computing Machinery, New York, NY, USA (2017). 10.1145/3123266.3123394

[4] Vasileva, M.I., Plummer, B.A., Dusad, K., Rajpal, S., Kumar, R., Forsyth, D.: Learning type-aware embeddings for fashion compatibility. In: Ferrari, V., Hebert, M., Sminchisescu, C., Weiss, Y. (eds.) Computer Vision - ECCV 2018, pp. 405–421. Springer, Cham (2018)

[5] Tan, R., Vasileva, M., Saenko, K., Plummer, B.: Learning similarity conditions without explicit supervision. In: 2019 IEEE/CVF International Conference on Computer Vision (ICCV), pp. 10372–10381 (2019). 10.1109/ICCV.2019.01047

[6] Chen, W., Zhao, B., Huang, P., Xu, J., Guo, X., Guo, C., Sun, F., Li, C., Pfadler, A., Zhao, H.: Pog: Personalized outfit generation for fashion recommendation at alibaba ifashion, pp. 2662–2670 (2019). 10.1145/3292500.3330652

[7] Lin, Y.-L., Tran, S., Davis, L.S.: Fashion outfit complementary item retrieval. In: Proceedings of the IEEE/CVF Conference on Computer Vision and Pattern Recognition, pp. 3311–3319 (2020)

[8] Blondel, V., Guillaume, J.-L., Lambiotte, R., Lefebvre, E.: Fast unfolding of communities in large networks. Journal of Statistical Mechanics Theory and Experiment **2008** (2008). 10.1088/1742-5468/2008/10/P10008

[9] Cheng W-H, Song S, Chen C-Y, Hidayati SC, Liu J (2021). Fashion meets computer vision: a survey. ACM Comput. Surv. (CSUR).

[10] Jiang, W., Liu, S., Gao, C., Cao, J., He, R., Feng, J., Yan, S.: Psgan: Pose and expression robust spatial-aware gan for customizable makeup transfer. In: Proceedings of the IEEE/CVF Conference on Computer Vision and Pattern Recognition, pp. 5194–5202 (2020)

[11] Dong, H., Liang, X., Shen, X., Wu, B., Chen, B.-C., Yin, J.: Fw-gan: Flow-navigated warping gan for video virtual try-on. In: Proceedings of the IEEE/CVF International Conference on Computer Vision, pp. 1161–1170 (2019)

[12] Zhu, H., Cao, Y., Jin, H., Chen, W., Du, D., Wang, Z., Cui, S., Han, X.: Deep fashion3d: A dataset and benchmark for 3d garment reconstruction from single images. In: European Conference on Computer Vision, pp. 512–530 (2020). Springer

[13] Tiwari, G., Bhatnagar, B.L., Tung, T., Pons-Moll, G.: Sizer: A dataset and model for parsing 3d clothing and learning size sensitive 3d clothing. In: Computer Vision–ECCV 2020: 16th European Conference, Glasgow, UK, August 23–28, 2020, Proceedings, Part III 16, pp. 1–18 (2020). Springer

[14] Ge, Y., Zhang, R., Wang, X., Tang, X., Luo, P.: Deepfashion2: A versatile benchmark for detection, pose estimation, segmentation and re-identification of clothing images. In: Proceedings of the IEEE/CVF Conference on Computer Vision and Pattern Recognition, pp. 5337–5345 (2019)

[15] Liu, Z., Yan, S., Luo, P., Wang, X., Tang, X.: Fashion landmark detection in the wild. In: European Conference on Computer Vision, pp. 229–245 (2016). Springer

[16] Gong, K., Liang, X., Zhang, D., Shen, X., Lin, L.: Look into person: Self-supervised structure-sensitive learning and a new benchmark for human parsing. In: Proceedings of the IEEE Conference on Computer Vision and Pattern Recognition, pp. 932–940 (2017)

[17] Liao, L., He, X., Zhao, B., Ngo, C.-W., Chua, T.-S.: Interpretable multimodal retrieval for fashion products. In: Proceedings of the 26th ACM International Conference on Multimedia, pp. 1571–1579 (2018)

[18] Wang, X., Wu, B., Zhong, Y.: Outfit compatibility prediction and diagnosis with multi-layered comparison network. In: Proceedings of the 27th ACM International Conference on Multimedia, pp. 329–337 (2019)

[19] Dong, X., Song, X., Feng, F., Jing, P., Xu, X.-S., Nie, L.: Personalized capsule wardrobe creation with garment and user modeling. In: Proceedings of the 27th ACM International Conference on Multimedia, pp. 302–310 (2019)

[20] Yin, W., Fu, Y., Ma, Y., Jiang, Y.-G., Xiang, T., Xue, X.: Learning to generate and edit hairstyles. In: Proceedings of the 25th ACM International Conference on Multimedia, pp. 1627–1635 (2017)

[21] Heilbron, F.C., Pepik, B., Barzelay, Z., Donoser, M.: Clothing recognition in the wild using the amazon catalog. In: ICCV Workshops, pp. 3145–3148 (2019)

[22] Ma, Y., Yang, X., Liao, L., Cao, Y., Chua, T.-S.: Who, where, and what to wear? extracting fashion knowledge from social media. In: Proceedings of the 27th ACM International Conference on Multimedia, pp. 257–265 (2019)

[23] Wu, B., Cheng, W.-H., Liu, P., Liu, B., Zeng, Z., Luo, J.: Smp challenge: An overview of social media prediction challenge 2019. In: Proceedings of the 27th ACM International Conference on Multimedia, pp. 2667–2671 (2019)

[24] Celikik, M., Kirmse, M., Denk, T., Gagliardi, P., Mbarek, S., Pham, D., Ramallo, A.P.: Outfit generation and recommendation–an experimental study. In: Dokoohaki, N., Jaradat, S., Corona Pampín, H.J., Shirvany, R. (eds.) Recommender Systems in Fashion and Retail, pp. 117–137. Springer, Cham (2021)

[25] He, K., Zhang, X., Ren, S., Sun, J.: Deep residual learning for image recognition. CoRR arXiv:1512.03385 (2015)

[26] Sanh, V., Debut, L., Chaumond, J., Wolf, T.: Distilbert, a distilled version of BERT: smaller, faster, cheaper and lighter. CoRR arXiv:1910.01108 (2019)

[27] Devlin, J., Chang, M.-W., Lee, K., Toutanova, K.: BERT: Pre-training of deep bidirectional transformers for language understanding. In: Proceedings of the 2019 Conference of the North American Chapter of the Association for Computational Linguistics: Human Language Technologies, Volume 1 (Long and Short Papers), pp. 4171–4186. Association for Computational Linguistics, Minneapolis, Minnesota (2019). 10.18653/v1/N19-1423. https://aclanthology.org/N19-1423

[28] Sarkar, R., Bodla, N., Vasileva, M.I., Lin, Y.-L., Beniwal, A., Lu, A., Medioni, G.: OutfitTransformer: Learning Outfit Representations for Fashion Recommendation. arXiv (2022). 10.48550/ARXIV.2204.04812

[29] Hu Z-H, Li X, Wei C, Zhou H-L (2019). Examining collaborative filtering algorithms for clothing recommendation in e-commerce. Text. Res. J..

